# Force and power requirement for development of cumin harvester: a dynamic approach

**DOI:** 10.1038/s41598-024-64473-y

**Published:** 2024-06-13

**Authors:** Mohit Kumar, Pramod Kumar Sahoo, Dilip Kumar Kushwaha, Indra Mani, Nrusingh Charan Pradhan, Abhishek Patel, Aqil Tariq, Sajid Ullah, Walid Soufan

**Affiliations:** 1 Sri Karan Narendra Agriculture University, Jobner-Jaipur, 303329 India; 2 ICAR- Indian Agricultural Research Institute, New Delhi, 110012 India; 3 Vasantrao Naik Marathwada Krishi Vidyapeeth, Parbhani, 431402 India; 4 Department of Farm Machinery and Power Engineering, College of Agricultural Engineering and Post Harvest Technology, CAU Imphal, 737135 India; 5 Department of Wildlife Fisheries and Aquaculture, College of Forest Resources, Mississippi State University, Mississippi State, MS 39762-9690 United States; 6 Department of Water Resources and Environmental Engineering, Nangarhar University, Jalalabad, 2600 Nangarhar Afghanistan; 7 Plant Production Department, College of Food and Agriculture Sciences, King Saud University, 11451 Riyadh, Saudi Arabia

**Keywords:** Cutting force, Power, Cumin, Blade type, Dynamic conditions, Plant sciences, Environmental sciences, Engineering

## Abstract

An experimental setup was developed for simulating the field conditions to determine the force and power required for cutting cumin crops in dynamic conditions. The effect of cutter bar speeds, forward speeds, and blade type on cutting force and power requirement for cutting cumin were also studied. Experiments were carried out at three levels: cutter bar speeds, forward speeds, and blade type. The results showed that all the factors significantly affected cutting force. The cutting force followed a decreasing trend with the increase in cutter bar speed. Whereas it followed an increasing trend with the increase in forward speed. The maximum cutting force for all three blades was observed at a cutter bar speed of 2.00 strokes.s^-1^ and forward speed of 0.46 m.s^−1^. The idle power and actual power required for cutting the cumin crop were also determined based on the cutting force. The results obtained were validated by the power drawn from the power source while operating the cutter bar blades. The R^2^ values for Blade-B1, Blade-B2, and Blade-B3 were 0.90, 0.82, and 0.88, respectively. The cutting force was primarily affected by the cutter bar speed, resulting in PCR values of 74.20%, 82.32%, and 81.75% for Blade-B1, Blade-B2, and Blade-B3, respectively, followed by the forward speed, which also had an impact on PCR values of 16.60%, 15.27%, and 18.25% for Blade-B1, Blade-B2, and Blade-B3, respectively. The cutting force for Blade-B1, Blade-B2, and Blade-B3 varied from 15.96 to 58.97 N, 21.08 to 76.64 N, and 30.22 to 85.31, respectively, for the selected range of cutter bar speed and forward speed. Blade-B1 had 18 and 30% less power consumption than Blade-B2 and Blade-B3, respectively.

## Introduction

Cumin (Cuminum cyminum L.) crop is a small annual herbaceous plant^1,2^. The cumin plant is short in height i.e., 20 to 50 cm^3^. In most parts of India, the crop matures between the months of February and the end of March. It is harvested manually using conventional tools like a sickle. The manual method of harvesting is time-consuming, labour-intensive, and comes under the moderately heavy work category^4^. Therefore, there is an urgent need to design and develop a harvesting machine to address the above problems. Hence, to design a harvesting machine with appropriate operational parameters, information regarding plant properties and energy required to cut the cumin crop is necessary^5^. The cutting process is an inevitable part of the harvesting machine. In order to cut the crop effectively with minimum losses, it is essential to select a suitable cutter bar and its power source. The crop's cutting force and power requirement help in selecting the suitable cutter bar and efficient power source^6,7^. The power source is decided based on the maximum power required to cut the plants effectively with minimum wastage.

Thus, it is necessary to measure the cutting force and power required for cutting cumin crops and their dependency on the various crop and machine parameters. A suitable harvester can be developed that minimizes losses and optimizes harvesting efficiency by quantifying the factors that impact the cutting force and power requirements.

Extensive work has been done by different researchers to determine the cutting energy required to cut different crops such as sorghum stalk (7.87–12.55 N.m), pigeon pea stem (20–190 N.m), and maize stalk (peak cutting force of 272.4 N)^8–10^. The cutting energy of a plant stem can be estimated from the relationships between the cutting force and the displacement of the knife (force–displacement curves). Thus, cutting force is the most important parameter for determining the energy required for cutting the crop.

The cutting force or power required to cut crop stem is dependent on various parameters such as moisture content (10–80%), stem diameter (3–25 mm), type of crop, crop variety, knife speed (0.5–2.0 m.s^−1^), knife type (serrated and flat edge) and feed rate (10–100 mm.min^−1^)^5,10–17^. It can be determined by two methods i.e., static shear test and dynamic shear test^15^. These methods were used in various studies to determine the cutting force and cutting energy of different plant stems^10^. However, the static shear test method was used in most of the studies. It was determined by using the universal testing machine, impact-type pendulum testing rig, texture analyzer, and static shear test apparatus. Various researchers had determined cutting force or cutting energy by static method for different crop stems such as sorghum stalk (34.10–142.70 mJ.mm^−2^), alpha stem (20.20–345.80 mJ), soybean (191.09–270.66 N), cumin stem (5.06–53.07 N), rose flower (5.97–9.99 N), paddy stem (11.86–25.48 N), grape cane (234.50–303.80) and cassava stem (18.20–25.60 kJ.m^−2^)^6,16–22^.

Most cutting force determination experiments were performed using a shear test rig or an impact-type pendulum testing rig^6^. These methods were used to measure static cutting force. However, these methods may not accurately describe the cutting action in dynamic motion, such as cutting using reciprocating knives.

The dynamic shear test method was also used by a few researchers to determine cutting force and cutting energy for finger millet, rice stem, bengal gram, cabbage stem, and onion leaves^7,15,23–25^. Tabatabaei and Borgheei^15^ and Nisha and Saravanakumar^23^ developed an experimental setup for measuring cutting force at reciprocating cutter bar with the help of load cell while cutting plant stems. The similar set was also used by Ramachandran and Ashokan^24^ to determine the cutting energy of Bengal gram and effect of stem diameter, moisture content, cutter bar speed and stroke length on cutting energy.

Sahoo and Raheman^26^ also developed a model that could estimate the required torque and power to cut paddy crop the effect of stem cross-section area, knife speed, and feed rate on cutting torque and power were also considered. A similar study was also conducted by Modak and Raheman^27^ on the same crop to study the effect of cutting speed, forward speed, and cutting stroke on cutting force. Kumawat and Raheman^25^ also determined the cutting torque required for topping onion leaves at different cutting widths, cutting speeds, and forward speeds. Similarly, Sarkar and Raheman^7^ developed an experimental setup for determining cutting torque for cabbage stems at different cutting positions, cutting speeds, and forward speeds.

However, limited study is available on the cutting force required to cut cumin stem. Mahmoodi et al.^21^ conducted the study on cumin stem for determination of cutting force. The study was conducted for the Iranian cumin variety, and the static shear force was determined using a UTM machine. The static shear test may not represent the cutting power required for the cumin harvester in actual field conditions. Therefore, this study was planned to determine the cutting force and power required to cut cumin stems for Indian varieties using the dynamic shear test method while simulating the actual field conditions. Hence, an experimental setup was developed to measure cutting forces at different cutter bar speeds, forward speeds, and blade types.

## Material and methods

### Selected test materials and location of study (samples)

The cumin variety GC-04 was selected for the study. The GC-04 variety is very popular among farmers because of its high-yielding capacity and resistance to major diseases of cumin, such as fusarium wilt, powdery mildew, and Alternaria blight^28–30^. The crop was planted at the National Research Centre on Seed and Spices (NRCSS), Ajmer, Rajasthan (test site, farm), during the main crop season in 2019–2020. NRCSS is located 26º36’ N latitude 75º49’ E longitude. The crop was planted in October by a conventional seed drill, which was modified for seed and spices and harvested in March by manually uprooting the plants after 120 days of sowing. The soil type was sandy loam, with a bulk density of 1550–1650 kg.m^−3^.

**Figure 1:**
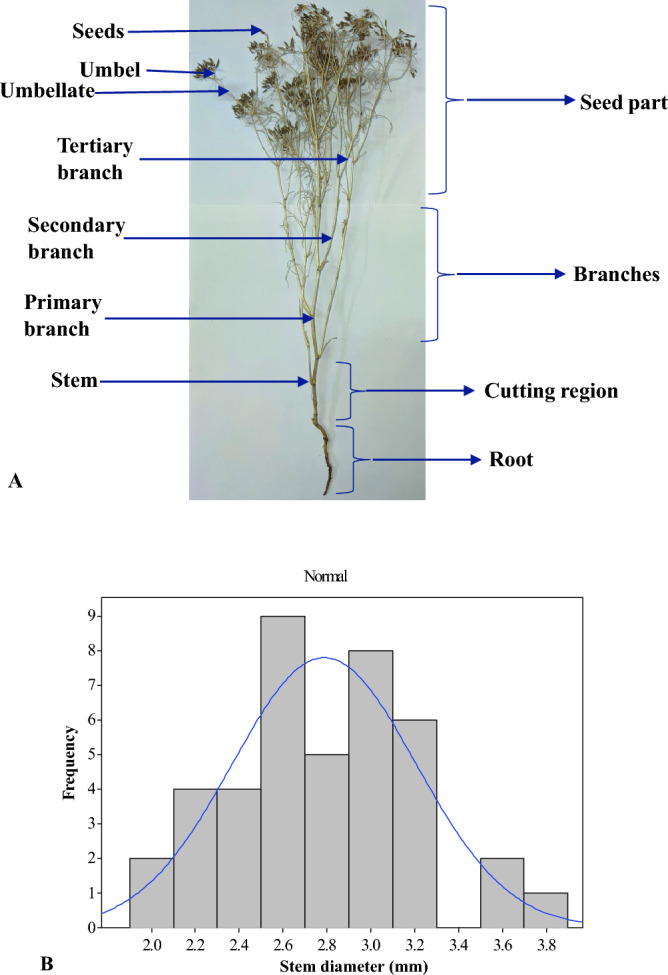
Cumin plant sample, (**A**) Marking for the uprooted cumin plant, (**B**) Stem diameter.

A sample size of 150 plants was selected randomly from the experimental field. The height of the selected plant should lie between 250 and 350 mm. Fully matured and healthy plants were considered for the study. The plants contaminated with pesticides or other toxins were discarded. The stem diameter of each plant was measured using a Vernier caliper (least count of 0.01 mm). It was measured at a height of 5 cm from the ground surface. A mark was placed around the plant's stem near the ground surface before uprooting the plant (Fig. 1A). This mark was taken as a reference point for measuring the height from the ground surface. Cumin plants were harvested manually by uprooting them along with their roots. During the uprooting of the plant, care was taken to avoid physical damage to the plant. The selected plant samples were packed in poly bags and cartons to avoid physical damage to the stems. The plant samples were transported to the laboratory at the Indian Agricultural Research Institute, New Delhi. The average stem diameter of the uprooted plant sample was 2.79 ± 0.42 mm for 150 randomly collected samples. The obtained data was distributed in the range of 2.0 to 3.8 mm, as depicted by the histogram (Fig. 1B).

### Experimental setup

**Figure 2:**
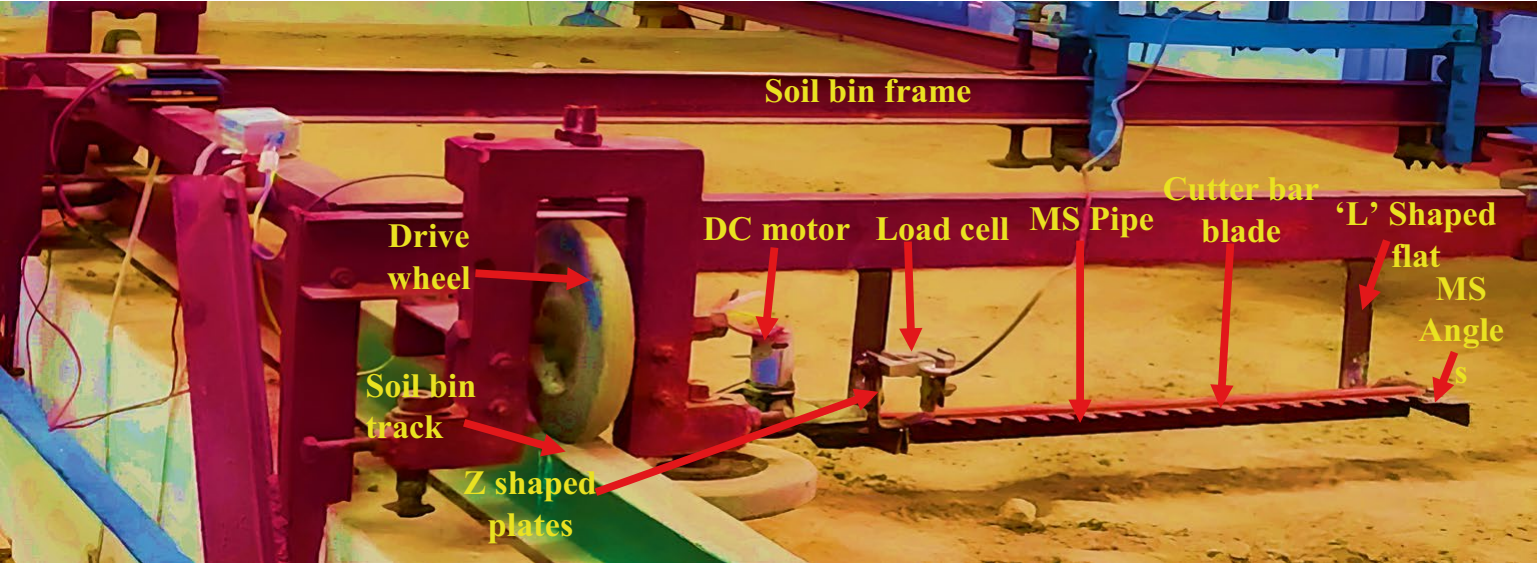
Experimental setup for cutting force measurement by dynamic method.

The cutting force by the dynamic method was determined with the help of a developed experimental setup. The setup consists of cutter bar blades (double-acting type), a DC motor (RS-775, DC, 18 V, 1989.30 rad.s^−1^), a battery (Li-ion, 20 V, 2 Ah), a motor controller (50 V and 10 A), an Arduino (ATmega-2560) board, a load cell (S-type, 20 kg, 0.01% sensitivity), and a rectangular frame of MS material (Fig. 2).

**Figure 3:**
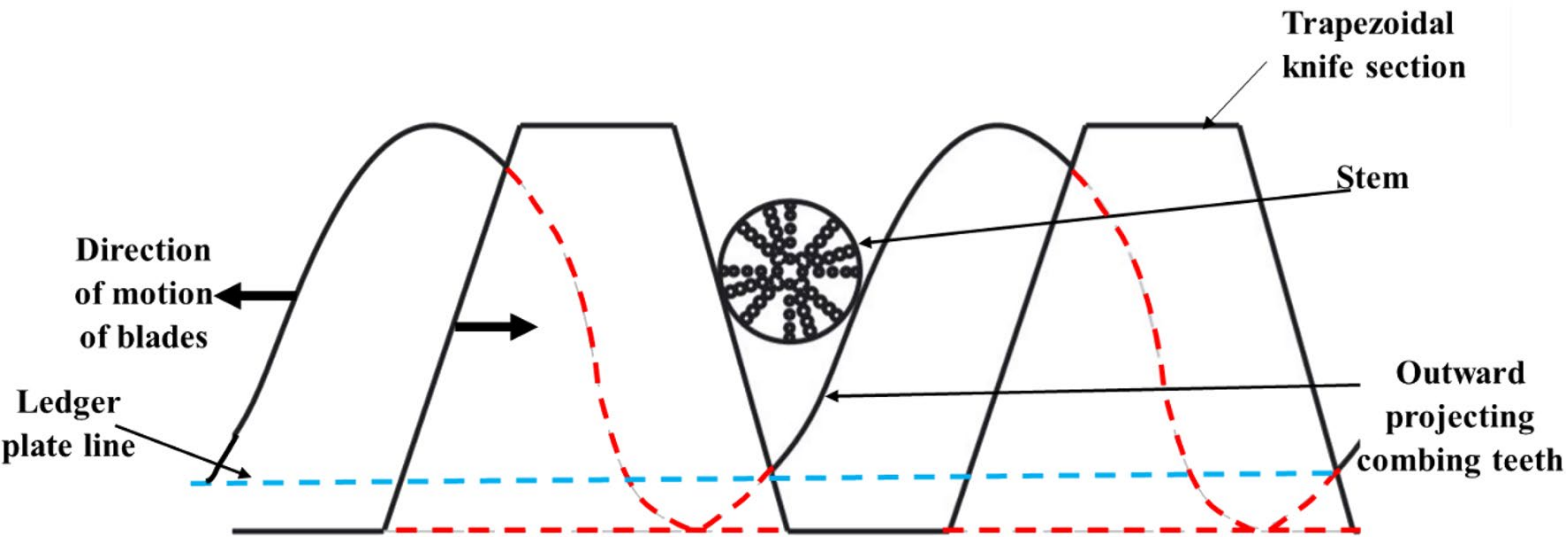
Knife section of the cutter bar blade.

On one side of the blade was a trapezoidal section, and on the other, there were outward-projecting combing teeth (curved profile section, as shown in Fig. 3). Both blades moved simultaneously in opposite directions, with a phase difference of π.

**Figure 4:**
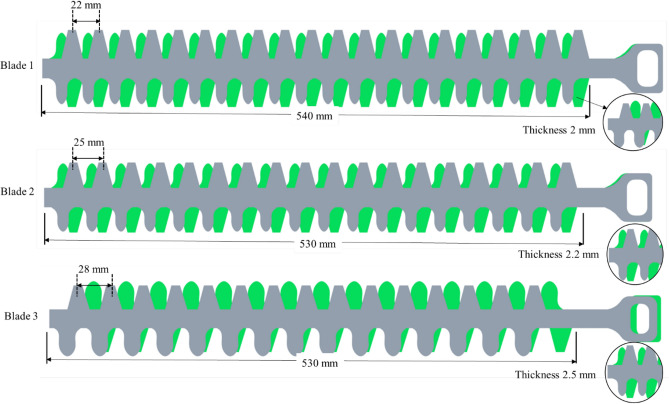
Three types of cutter bar blades i.e., (**A**) Blade-B1, (**B**) Blade-B2, and (**C**) Blade-B3.

Three double acting reciprocating cutter bar blades of different bevel angle and pitch were selected to determine the effect of blade type on cutting force Fig. 4A–C. The double-acting blade was selected because it has the least vibration, as suggested by Huang et al.^31^.

The cutter bar was operated by a 150 W DC motor, and the eccentric cam and follower mechanism converted the motor's rotary motion into linear motion. The cutter bar was mounted on the frame. Table 1 gives a detailed description of the selected blades.

**Table 1:** Detail specification of selected cutter bar blades.

Sl. no	Particulars	Specifications		
		Blade-B1	Blade-B2	Blade-B3
1	Type of cutter bar	Reciprocating double acting	Reciprocating double acting	Reciprocating double acting
2	Length of cutter bar	550 mm	530 mm	530 mm
3	Knife section	Standard	Standard	Standard
4	Blade	Plain	Plain	Plain
5	Thickness of blade	2 mm	2.2 mm	2.5 mm
6	Knife material	0.7–0.95% C 0.3–0.5% Mn	0.7–0.95% C 0.3–0.5% Mn	0.7–0.95% C 0.3–0.5% Mn
7	Hardness	48 HRC	55 HRC	58 HRC
8	Blade	Plain	Plain	Plain
9	Knife profile	Trapezoidal and curved profile having a radius 4 mm	Trapezoidal and curved profile having a radius 5.5 mm	Trapezoidal and curved profile having a radius 6.5 mm
7	Pitch	22 mm	25 mm	28 mm
8	Clearance between knife section	0.1 mm	0.12 mm	0.13 mm
9	Material	High carbon steel	High carbon steel	High carbon steel
10	Bevel angle	22.5°	25°	28°

In order to determine the cutting force, an S-type load cell of a capacity of 20 kg was mounted on the cutter bar. A slot of 5 mm clearance was cut on the upper blade of the cutter bar to mount the load cell. Two ‘Z’ shaped steel flats were welded on both sides of the cut slot. The load cell was fixed between these two ‘Z’ shaped plates with the help of nuts and bolts (Fig. 2).

The whole assembly was mounted on the soil bin. The forward speed was varied with the help of a soil bin trolley. A frame of MS square section pipe having 600 mm length was fabricated to mount the cutter bar assembly on the soil bin. The cross-section area and thickness of the pipe were 25 × 25 mm and 2.0 mm, respectively. Two pieces of MS angle of size 25 × 25 mm having 100 mm length were welded on both ends of the pipe. The cutter bar was fixed between these two MS angles. After that, this assembly was attached to the soil bin platform with the help of two flats of size 40 × 5 × 2 mm^3^ folded in L shape at a length of 10 mm (Fig. 2).

### Electrical connections

**Figure 5:**
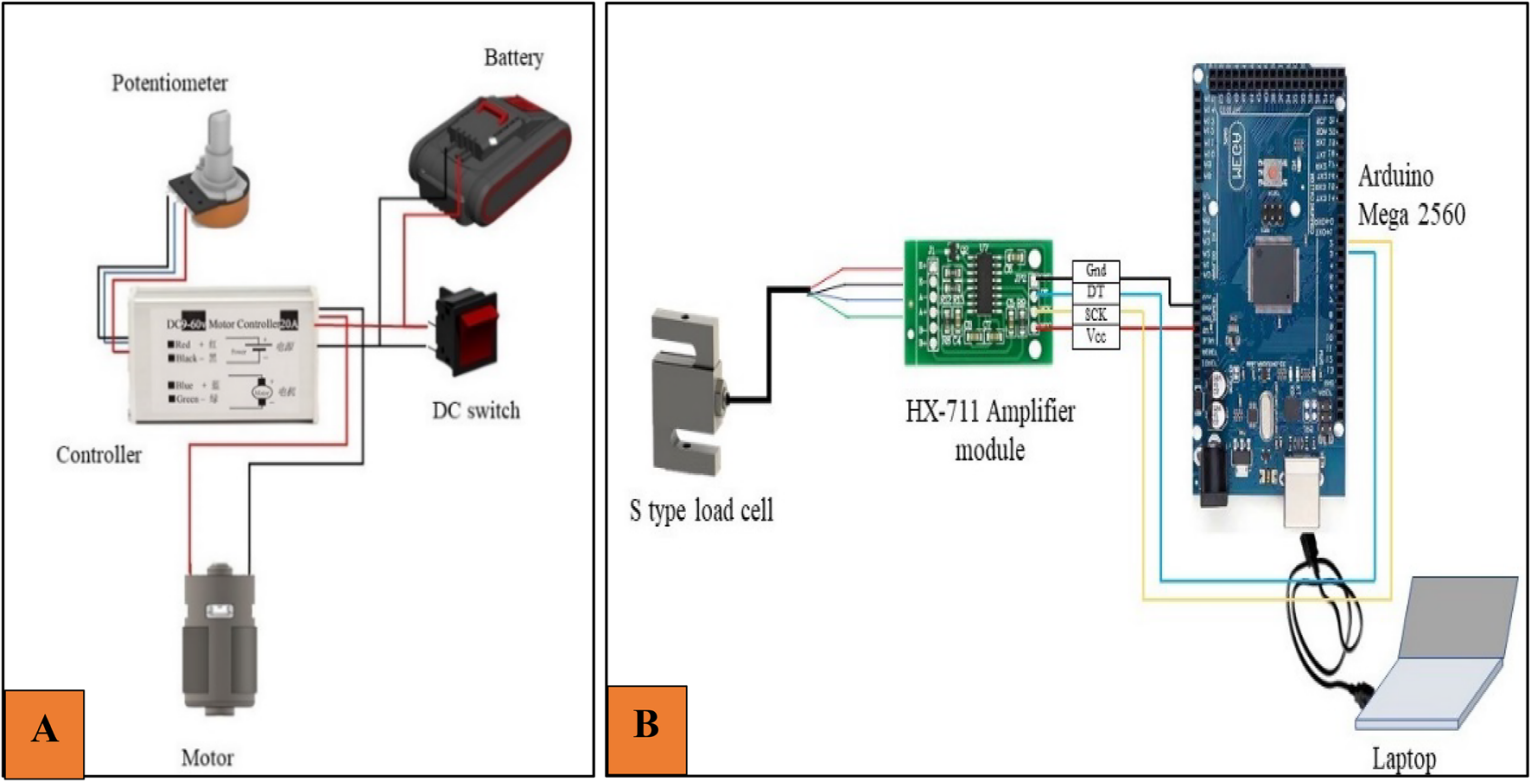
Schematic representation of cutter bar force measurement by dynamic method, (**A**) Operation of cutter bar (**B**) Measurement of force by load cell.

The cutter bar was operated by a DC motor and the required speeds of the motor were achieved by changing the voltage using a motor controller. The schematic representation of cutter bar speed control is shown in Fig. 5A. The load cell was connected with a 24-bit HX711 ADC module, and then the signal was taken through Arduino (ATmega 2560) to the computer, and the data was recorded with the help of a serial oscilloscope (Fig. 5B). Serial oscilloscope (HTC make, PDO-5025S Model) having Y deflection of 2mv.div^−1^ to 50v.div^−1^ ensures precise signal analysis across a broad spectrum of amplitudes. In order to install the oscilloscope, it is placed on a stable surface, connected with a power source and any required probes, and then a serial connection is established between the computer and the oscilloscope via USB or Ethernet. Serial monitor software is installed to establish communication between the oscilloscope and the computer. Once powered on, trigger settings must be configured for stable waveform acquisition, vertical and horizontal settings are adjusted for optimal display, and data acquisition is initiated manually or through automated sequences. The data can be saved and captured to the internal memory of the connected computer for further analysis.

### Anchoring of plants

**Figure 6:**
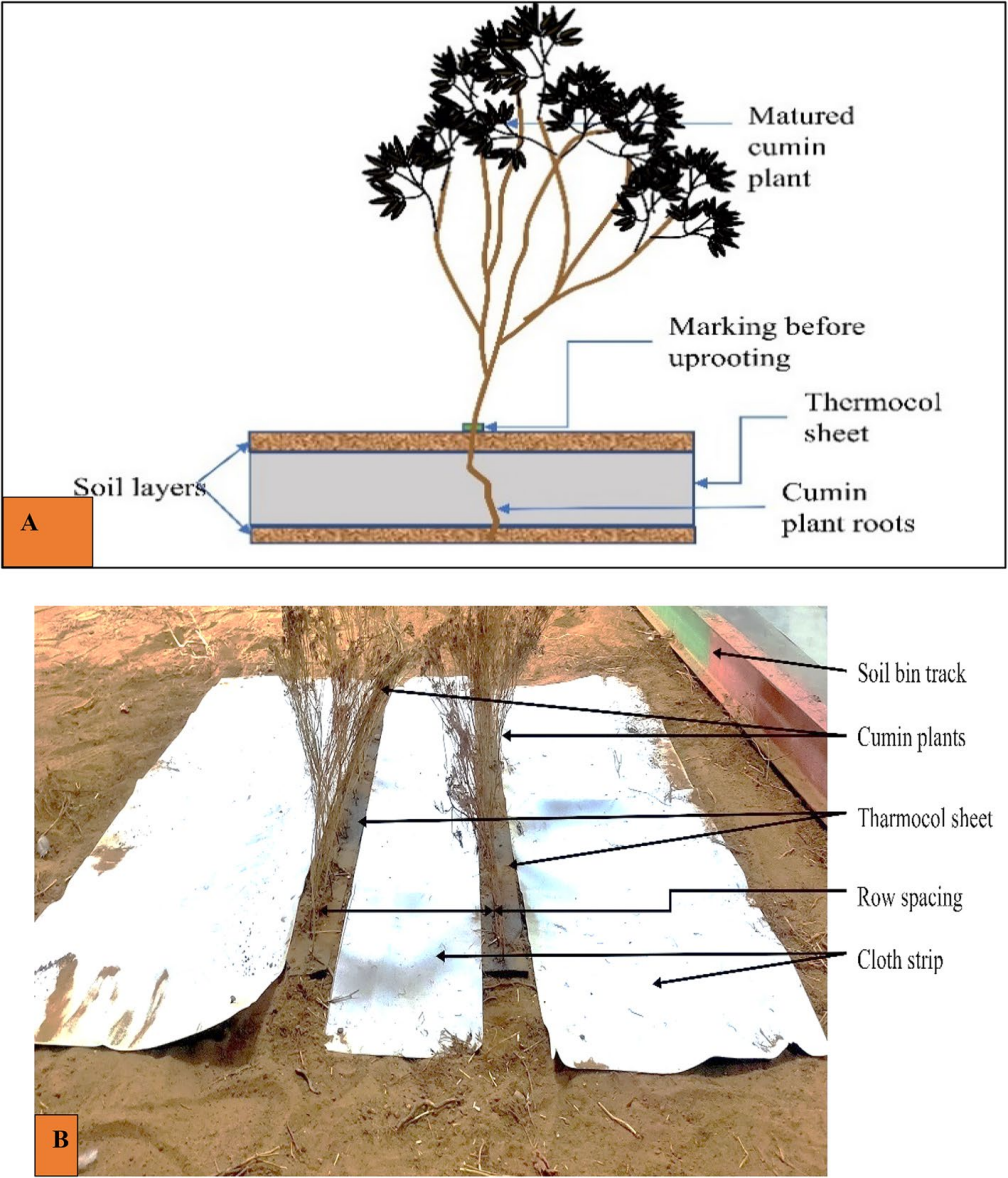
(**A**) Placement of cumin plant for anchoring to simulate field condition (**B**) Anchored cumin plants on soil bin.

In order to simulate the field conditions, the plants were anchored in the soil bin. The plants were anchored such that the roots remained inside this thermocol sheet. The plants were inserted inside the thermocol sheet up to the marked point in the standing position (Fig. 6A). Therefore, two thermocouple blocks of size 500 × 100 × 10 mm were inserted inside the soil surface in parallel positions at a distance of 25 cm (i.e., recommended row spacing of cumin plants) apart for anchoring the plants in the soil bin (Fig. 6B). The plants were picked randomly from the experimental field. The marked point was taken as a reference point for anchoring the plant (Fig. 1A).

### Experimental design

The study was conducted to see the effects of the cutter bar blade, cutter bar speeds, and forward speeds on cutting force for the cumin plant. Face-centered central composite design (CCD) was used to study the effect of blade type, cutter bar speeds, and forward speeds on the cutting force. Three levels of each independent parameters were selected for the study (Table 2). Three double acting cutter bar blades of different bevel angles and pitch were selected to study blade type's effect on cutting force. In normal cutting action, the stem should be pinched between the cutting edges of the knife section. The condition for the stem to be clamped by a double-acting cutter bar is satisfied when the cutting angle of the blade is less than the friction angle between the blade and the stem^31^. The friction angle between the cumin stem and blade was 23.7°^21^. Therefore, the blades having cutting angle of the range 22.5 to 28 degree (based on market availability) were selected.

**Table 2:** Experimental plan for measuring the cutting force of cumin crop.

Parameters	Levels	Values
Blade type	3	Blade-B1, Blade-B2 and Blade-B3
Cutter bar speed (strokes/s)	3	2.00, 12.50 and 18.30
Forward speed (m/s)	3	0.13, 0.30 and 0.46
Crop variety	1	GC-04
Dependent parameter		
Cutting force (N)		

Cutter bar speed was measured by recording the frequency of the cutter bar using a Serial Oscilloscope. The forward speed of the soil bin trolley was measured by recording the distance travelled per unit time at different gear combinations of the soil bin^32^. Preliminary trials were conducted to find the minimum cutter bar speed required for smooth cutting of cumin plants, and it was determined to be 2.00 strokes.s^−1^. During the experiment the shattering of cumin plant was observed more than 2% at cutter bar speed of 18.30 strokes/s. As per BIS, 2% cutter bar losses are permissible^33^. Hence, the maximum cutter bar speed was selected as 18.30 strokes/s. The forward speed was limited between 0.13 to 0.46 m.s^−1^ in the experimental plan to facilitate the operational speed of walk-behind harvesters (0.5 to 2.2 km.h^−1^)^25,26,34,35^. A total of 29 experimental combinations for each blade type were obtained by the “Design-Expert 13.0” software using face-centered CCD design, and three replications for each treatment with five center points were carried out using the developed experimental setup.

### Test procedure

**Figure 7:**
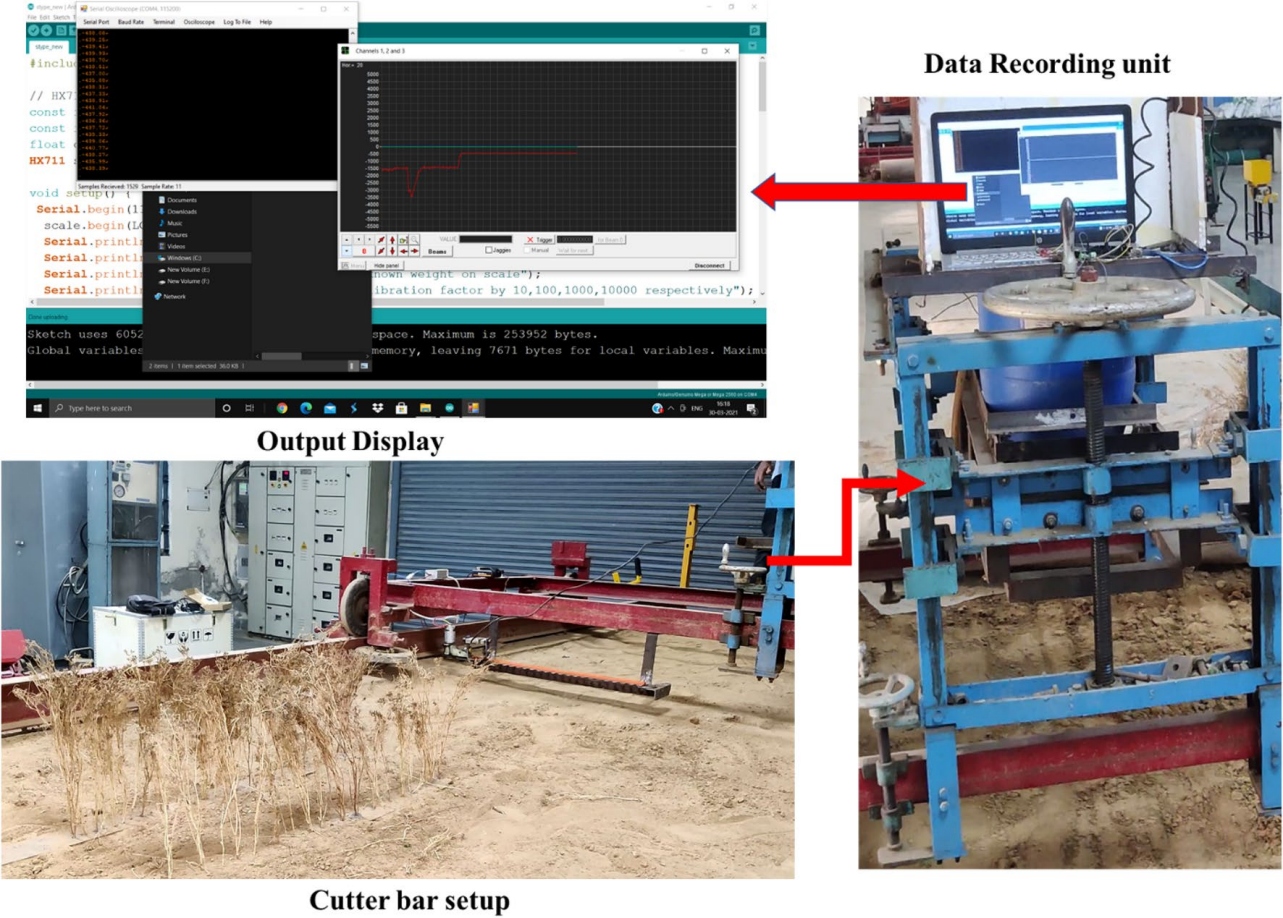
Cutting force measurement by developed experimental setup.

After the development of the experimental setup, plant samples were selected randomly from the bag, and the moisture content of samples was determined using the oven-dry method following the procedure described by Pathak et al.^36^. The moisture content of the cumin plants was observed to be 17.23 ± 20% (w.b.) during the experiment. The experiments were conducted as per the experimental plan (Table 2). The reciprocating cutter bar blade cuts the anchored plants as the soil bin trolley moves. Subsequently, the signal from the load cell was recorded using the software “serial oscilloscope” in the “.csv” file in terms of grams. The obtained output in the form of grams was converted into Newton (Fig. 7).

The cutting force was calculated as the difference between the average readings for the load and no-load conditions. The data was recorded for the selected cutter bar speeds, forward speeds, and blade type. Each experiment was replicated thrice.

After that, the cutter bar power was calculated using the obtained cutting force values and cutter bar speed in Eq. (1)^37^ and Eq. (2)^26^. The cutter bar speed was determined using the stroke of the knives and the frequency of oscillations.

1 $$P_{m} = F_{c} \times \nu_{c}$$

2 $$P_{T} = (F_{c} + F_{i} ) \times \nu_{c}$$

where; P_m_ is the calculated power in cutting cumin plants (W), F_c_ is the cutting force (N), ‘F_i_’ is the force recorded when cutter bar is operated at no load condition (N) and ν_c_ is the cutter bar speed (m/s).

The power required to cut the plant material is the difference between the total power required for cutting and the idle power required to operate the cutting device (Eq. (3))^26^. The idle power losses can be eliminated by subtracting readings of no-load conditions from load conditions.

3 $$P_{m} = P_{T} - P_{i}$$

where: P_m_ is the calculated power in cutting (W), P_T_ is the total calculated power required in cutting (W), and P_i_ is the calculated idle power required in operating the cutter bar (W).

The calculated power obtained by Eq. (3)^26^ was validated with the observed power during the operation. The observed power was determined by the Eq. (4)^38^.

4 $$P = V \times I$$

where; P is the observed power, V is the voltage (V), and I is the current (A).

The voltage and current were measured with the help of a digital multi-meter (Accuracy of DC volt = 0.5% ± 3 V and DC current = 1.5% ± 3 A). The power requirement of the cutter bar Blade-B1, Blade-B2, and Blade-B3 was observed for load and no-load conditions. The power recorded during the no-load condition was the observed idle power. Whereas the power recorded at load condition is the total observed power required to cut the cumin plants. The results of calculated and observed power were compared and validated.

### Statistical analysis

The results were interpreted through statistical analysis using response surface methodology (RSM) in central composite design. This statistical analysis was performed using the “Design Expert -13” software. The face-centered central composite design (CCD) was used to understand the significance of variables, viz. cutter bar speed and forward speed interactions for each blade^26,39,40^. At first, to analyze cutting force, the selection of model was done based on the ANOVA table obtained from the software. The selected model was tested for adequacy. In order to test the adequacy of the model, the lack of fit test and coefficient of determination R^2^ were used. If the lack of fit is not significant, the model is adequate. Whereas, a higher R^2^ value indicates a better fit^41^. After selecting the best model, experimental data were fitted to the selected model to find the effect of each independent variable on the response and the relationship between independent variables and responses^42^. The diagnostics analysis of the applied model was checked using the predicted vs actual plot. After diagnostics analysis, the model was adequate to describe the effect of cutter bar speed, forward speed, and blade type on cutting force. Thus, the 3D surface graph is plotted between cutter bar speeds, forward speeds, and cutting force for each blade to find the effect of independent variables on the response at various points. Thereafter, maximum force and power required to cut the cumin stem for the three blades were compared using paired ‘t’ test to select suitable blade for cumin harvester.

### Experimental design

Experimental research and field studies on plants (either cultivated or wild), including the collection of plant material, must comply with ICAR- Indian Agricultural Research Institute New Delhi, 110012, India.

### Permission

We have permission to collect the cumin variety GC-04 from institute ICAR- Indian Agricultural Research Institute New Delhi, 110012, India.

## Results

### Cutting force

The average cutting force values for each blade are presented in Table 3. At first, model was selected for each blade to perform further analysis. Table 4 illustrates that the quadratic model was suggested for Blade-B1 and Blade-B2. Whereas a linear model was suggested for Blade-B3. Thereafter, the adequacy of the model was evaluated based on the lack of fit test and coefficient of determination. Table 5 showed that the lack of fit test of the selected model was not significant for all three blades, i.e., Blade-B1, Blade-B2, and Blade-B3. This showed that the models were adequate. The difference between the coefficient of determination (R^2^) and the adjusted coefficient of determination (adj. R^2^) was less than 0.20 (Table 4). It illustrated that there was an excellent correlation between the independent variable and the fitted model. Thus, the model can describe the independent variable adequately.

**Table 3:** Average cutting force at different combinations of cutter bar speed and forward speed for all three blades.

Cutter bar speed (strokes.s^−1^)	Forward Speed (m.s^−1^)	Cutting force (Blade-B1) (N)	Cutting force (Blade-B2) (N)	Cutting force (Blade-B3) (N)
2.00	0.13	44.60 ± 3.18	59.39 ± 2.31	65.66 ± 2.84
2.00	0.30	50.92 ± 1.36	68.54 ± 3.09	76.68 ± 1.69
2.00	0.46	56.74 ± 2.98	75.31 ± 1.39	84.51 ± 0.71
12.50	0.13	26.00 ± 2.42	42.88 ± 3.62	47.97 ± 3.40
12.50	0.30	27.09 ± 2.33	54.41 ± 5.10	60.25 ± 2.33
12.50	0.46	37.44 ± 3.41	64.90 ± 2.66	64.32 ± 2.08
18.30	0.13	17.19 ± 1.42	27.14 ± 5.38	33.44 ± 3.20
18.30	0.30	22.76 ± 1.94	32.45 ± 2.40	37.91 ± 1.60
18.30	0.46	31.73 ± 3.59	35.70 ± 1.85	48.76 ± 4.20

**Table 4:** Selection of model for Blade-B1, Blade-B2 and Blade-B3.

	Blade-B1			Blade-B2			Blade-B3		
Source	F-Value	p-value Prob > F	Remarks	F-Value	p-value	Remarks	F-Value	p-value	Remarks
Linear	11.75	< 0.0001		3.57	0.0144		1.98	0.116	Suggested
2FI	13.98	< 0.0001		3.64	0.0167		2.10	0.107	
Quadratic	1.58	0.2254	Suggested	2.52	0.087	Suggested	3.03	0.053	
Cubic	3.65	0.0705	Aliased	0.04	0.836	Aliased	5.68	0.027	Aliased
R^2^	0.96			0.95			0.97		
Adj. R^2^	0.95			0.94			0.96

**Table 5:** ANOVA for the effect of cutter bar speed, forward speed on cutting force for Blade-B1, Blade-B2 and Blade-B3.

Source	Blade-B1						Blade-B2						Blade-B3					
	SS	df	MSS	F-Value	p-value Prob > F	PCR (%)	SS	df	MSS	F-Value	p-value Prob > F	PCR (%)	SS	df	MSS	F-Value	p-value Prob > F	PCR (%)
C	3246.96	1	3246.96	445.55	< 0.0001**	74.2	5826.61	1	5826.61	382.39	< 0.0001**	82.32	5696.15	1	5696.15	677.08	< 0.0001**	81.75
ѵ_m_	726.85	1	726.85	99.74	< 0.0001**	16.60	1081.21	1	1081.21	70.96	< 0.0001**	15.27	1275.87	1	1275.87	151.66	< 0.0001**	18.25
C × ѵ_m_	4.27	1	4.27	0.59	0.4519	0.10	40.68	1	40.68	2.67	0.1159	0.58						
C^2^	371.19	1	371.19	50.93	< 0.0001**	8.50	122.20	1	122.20	8.02	0.0094**	1.73						
ѵ_m_^2^	35.76	1	35.76	4.91	0.037*	0.80	5.09	1	5.09	0.33	0.5688	0.07						
Residual	167.61	23	7.29				350.46	23	15.24				218.73	26	8.41			
Lack of Fit	32.12	3	10.71	1.58	0.2254		96.12	3	32.04	2.49	0.0872		81.54	6	13.59	1.98	0.1166	
Pure Error	135.49	20	6.77				254.34	20	12.72				137.19	20	6.86			
Cor Total	4587.05	28					7434.29	28					7190.75	28				

**Significant at 1% level of significance.

*Significant at 5% level of significance.

After selecting the best model, the independent variables were fitted in the selected model, and each independent variable's effect on the response was evaluated. The ANOVA was used to analyze the effect of cutter bar speed and forward speed on cutting force for Blade-B1, Blade-B2, and Blade-B3. ANOVA analysis in Table 5 showed that model terms ‘C’, ‘ѵ_m_’, ‘C^2^’ were significant at 1% level of significance and ‘ѵ_m_^2^’ was significant at 5% level of significance for Blade-B1. Whereas the interaction term C × ѵ_m_ was not significant. Song et al.^43^ and Vu et al.^44^ also reported that the linear and quadratic terms of the cutting speed significantly affected the cutting force. Table 5 showed that the cutting force for the Blade-B1 was predominantly affected by the cutter bar speed, with a percentage contribution ratio (PCR) of 74.20%. This indicates that the cutter bar speed accounted for the largest proportion of the observed variation in cutting force. After the cutter bar speed, cutting force was majorly affected by forward speed, with PCR values of 16.60%. The quadratic term of cutter bar speed and forward speed were also found to have significant effects on cutting force, with PCR values of 8.50% and 0.80% respectively. This implies that the quadratic term of these two parameters had lesser impacts on cutting force. Similarly, the model terms ‘C,’ ‘ѵm’, and ‘C^2^’were significant at a 1% level of significance in the case of Blade-B2, and the remaining terms (‘C × ѵm_’_ and ‘ѵ_m_^2^’) were non-significant^27^. Similar to Blade-B1, the cutting force was most significantly affected by the cutter bar speed, with PCR values of 82.32% for Blade-B2. The PCR values of forward speed and square term of cutter bar speed were 15.27 and 1.73% respectively. In case of Blade-B3, cutting force was most significantly affected by cutter bar speed (81.75%) and followed by forward speed (18.25%)^26,27^.

In order to develop a mathematical model to express the relationship between cutter bar speed and forward speed with cutting force, regression analysis was performed with the help of “Design-Expert 13.0” software for each blade^43,45^. Thus, after putting the estimated coefficient values of significant terms, equations for each blade were obtained. The model equation for Blade-B1, Blade-B2 and Blade-B3 are given in Eqs. (5), (6) and (7)^26^ respectively.

5 $${\text{C}}_{{\text{f1}} \, }\text{=} \, \text{68.59} \, - \, \text{5.62}\times {\text{C}} \, - \, \text{17.42}\times {\nu }_{\text{m}}\text{+} \, \text{0.15}\times {\text{C}}^{2}\text{+} \, \text{84.69}\times {\nu_{\text{m}}}^{2}$$

6 $${\text{C}}_{\text{f2}} \, \text{=} \, \text{50.61- 0.089 }\times \text{ C} \, \text{+} \, \text{84.26}\times \, {\nu}_{\text{m}}- \, \text{0.08 }\times \, {\text{C}}^{2}$$

7 $${\text{C}}_{\text{f3}} \, \text{=} \, \text{72.24} \, - \, \text{2.58}\times \text{ C} \, \text{+} \, \text{51.02}\times {\nu}_{\text{m}}$$

where; ‘C_f1_’ (N) is the cutting force for Blade-B1, ‘C_f2_’ (N) is the cutting force for Blade-B2, ‘C_f3_’ (N) is the cutting force for Blade-B3, ‘C’ is the cutter bar speed (strokes.s^-1^) and ‘ѵ_m_’ is the forward speed (m.s^−1^). The obtained coefficient of determination (R^2^) values for the Blade-B1, Blade-B2 and Blade-B3 were 0.96, 0.95, 0.97 respectively. The high values of R^2^ (> 0.95) indicates that the developed model can accurately explain the variability in the data^41,44^. The model equations are valid only for the given range of independent parameters.

### Steps for cutting force calculation using the equations

In order to calculate the cutting forces C_f1_, C_f2_ and C_f3_ using the given Eqs. (5), (6), and (7), follow these steps:

i. At first select the blade for which cutting force is to be determined
ii. Later select the equation accordingly
iii. Select the values of cutter bar speed and forward speed.
iv. Put the values of cutter bar speed and forward speed to the concerned equation.
v. Calculate the values.

**Figure 8:**
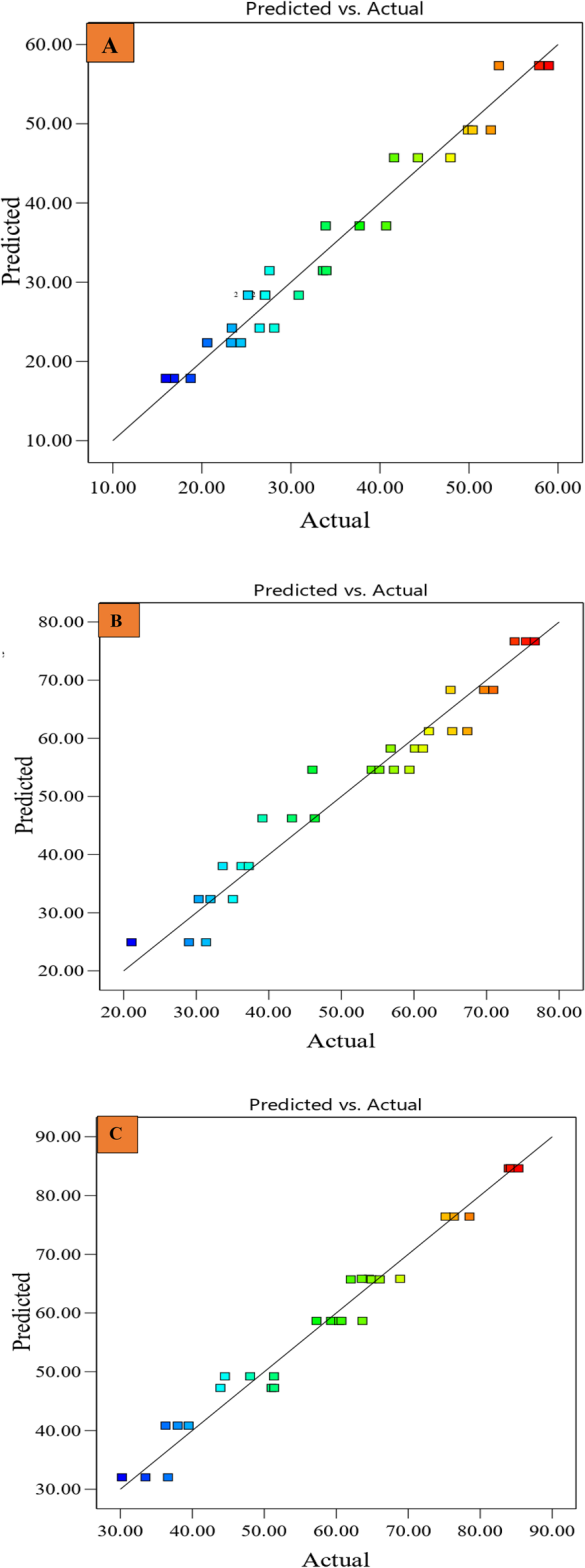
Predicted vs actual plot for (**A**) Blade-B1, (**B**) Blade-B2 and (**C**) Blade-B3.

Further, the variation in the experimental values and predicted values of all three blades, i.e., Blade-B1, Blade-B2, and Blade-B3, for the selected model presented by predicted v/s actual plot (Fig. 8A–C). In the predicted v/s actual graph, the predicted values from the regression model were compared with actual and experimental values^46^. Figure 8A–C showed that there was a good agreement between actual and predicted values, as the graph showed a linear relationship between predicted and actual values; also, the values were relatively close to each other^42^. This demonstrates that the models adequately describe the effect of different cutter bar speeds and forward speeds on cutting force for each blade.

### Effect of cutter bar speed and forward speed and cutter bar blade on cutting force

**Figure 9:**
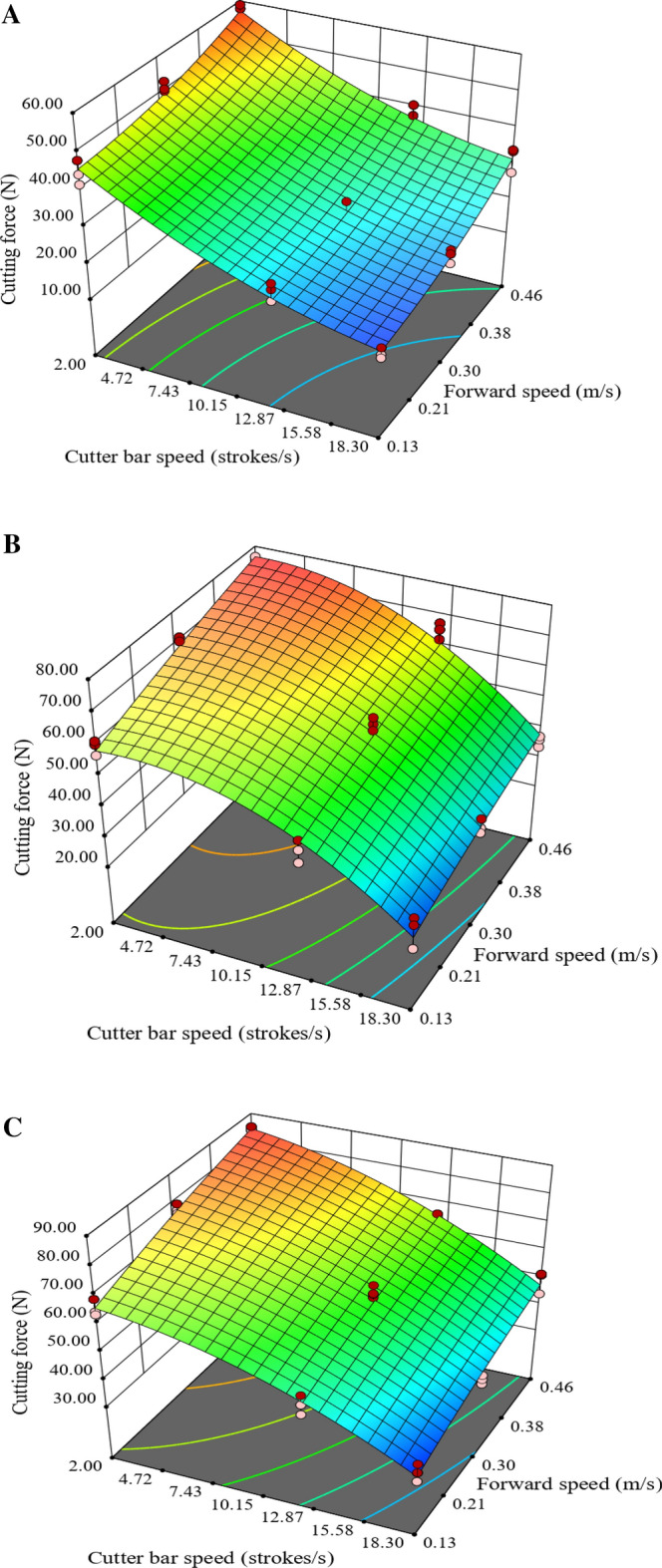
Effect of cutter bar speed and forward speed on cutting force (**A**) Blade-B1, (**B**) Blade-B2 and (**C**) Blade-B3.

After analysing the diagnostics of the model, the 3D surface graph (Fig. 9A–C) of each blade was plotted to analyse the effect of cutter bar speed and forward speed on cutting force for all the three blades. Figure 9 shows that the cutting force followed an increasing trend with the decrease in cutter bar speed for all three blades^26^. An increasing trend was observed with the increase in forward speed^47,48^. The maximum cutting force for Blade-B1, Blade-B2 and Blade-B3 was observed at cutter bar speed of 2.00 strokes.s^−1^ and forward speed of 0.46 m.s^−1^. The cutting force for Blade-B1 (Fig. 9A) at the fixed cutter bar speed of 18.30 strokes.s^−1^, increased from 15.96 to 31.44 N with the increase in forward speed (0.13 to 0.46 m.s^−1^). Whereas, at the cutter bar speed of 2.00 strokes.s^−1^, it varied from 45.32 to 58.97 N. In the case of fixing forward speed at 0.46 m.s^−1^, the cutting force decreased from 58.97 to 31.44 N with the increase in cutter bar speed (2.00 to 18.30 strokes.s^−1^). While at a forward speed of 0.13 m/s, it varied in the range of 15.96 to 45.32 N with the increase in cutter bar speed. Figure 9B revealed that the cutting force for Blade-B2 varied from 21.08 to 76.64 N for the selected range of cutter bar speed and forward speed. The results revealed that the maximum cutting force for Blade-B2 was 24.34% higher than that for cutter bar Blade-B1. The results of cutting force for Blade-B3 revealed that it varied from 30.22 to 85.31 N for the selected range of cutter bar speed and forward speed (Fig. 9C). The maximum cutting force for Blade- B3 was 44.66% and 11.32% higher than the cutting force for Blade B1 and Blade B2, respectively. It was also observed while determining the cutting force of the cutter bar Blade-B3 that the quality of the cut was not good, which might be due to improper clamping of the stem between knives as the cutting angle might be larger than the friction angle between the knife and stem for Blade-B3.

### Cutter bar power

After the cutting force analysis, the cutter bar power was calculated from the equations, i.e., Eqs. (2) and (3)^44^. The calculated power was also compared with the observed cutter bar power to validate the developed model. The calculated values of idle cutting power and the total cutting power required for the cumin crop were compared with the observed values using the predicted and actual graphs^49–52^.

#### Idle cutting power

**Figure 10:**
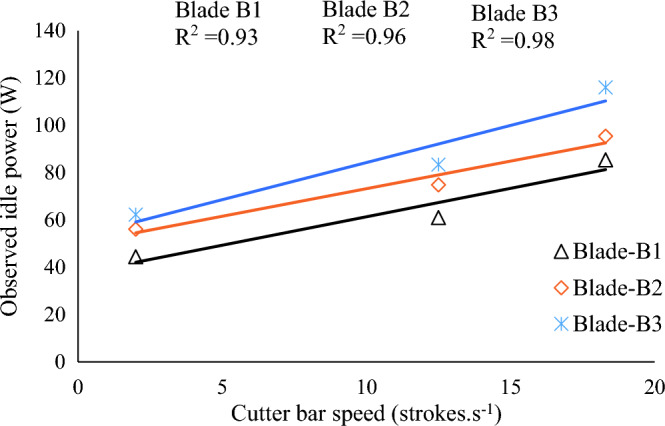
Effect of cutter bar speed on idle cutting power for Blade-B1, Blade-B2, and Blade-B3.

Figure 10 showed a good correlation between cutter bar speed and observed idle cutting power with the coefficient of determination value (R^2^) of 0.93, 0.96, and 0.98 for Blade-B1, Blade-B2, and Blade-3, respectively. The idle power followed a linearly increasing trend with the increase in cutter bar speed. This effect is because power increases as speed increases even if friction remains constant^26^. It was found that the idle cutter bar power recorded in no load condition for Blade-B1, Blade-B2, and Blade-B3 varied from 44.33 to 85.36 W, 56.04 to 95.27 W, and 62.18 to 115.90 W, respectively, with varying cutter bar speed from 2.00 to 18.30 strokes.s^-1^ (Fig. 10).

**Figure 11:**
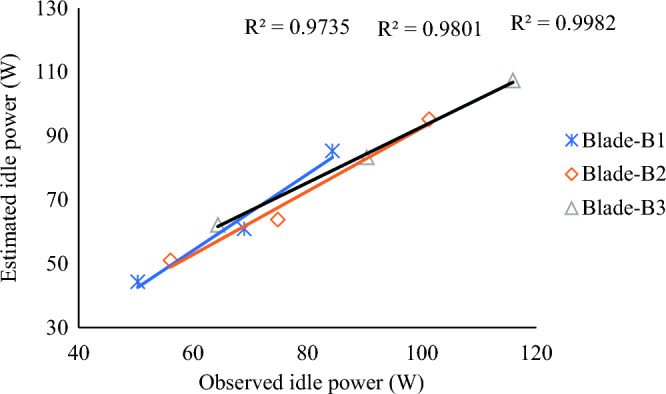
Comparative assessment of calculated and observed idle power for Blade-B1, Blade-B2 and Blade-B3.

The calculated values of the idle cutting power were plotted against the observed idle power. Figure 11 depicts that a good correlation was found between calculated and observed idle power values, with the coefficient of determination value (R^2^) values of 0.9735, 0.9801, and 0.9982 for Blade-B1, Blade-B2, and Blade-3, respectively. Thus, there was an adequate agreement between the calculated and observed power for the idle operation of the cutter bar.

#### Total cutting power

**Figure 12:**
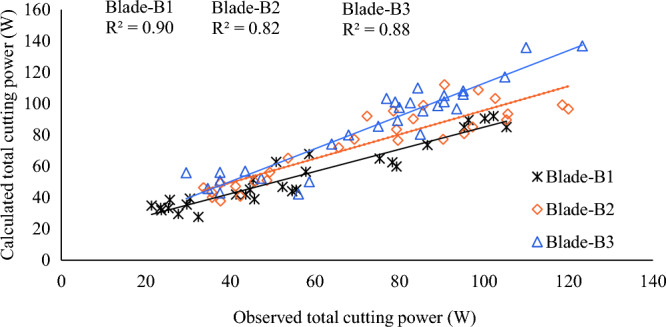
Comparison of calculated and observed total cutting power for Blade-B1, Blade-B2, and Blade-B3.

Similar to the idle power, the total power required for cutting the cumin crop for all three blades were also compared and validated. Figure 12 showed that there was a slight deviation in observed and calculated values of the total power required for all three blades in cutting cumin crop. The results revealed that the power requirements for all the three blades were nearly equal but at higher cutter bar speeds the power requirement had significant difference for each blade. The discrepancy between the calculated and observed values could be attributed to considering the average values of current and voltage instead of the area under the curve due to the limitation of the dynamic method of force determination. In addition, the vibration on the load cell caused by the cutter bar's frequency might also impact the readings. An excellent agreement between the calculated and observed values of total power for Blade-B1, Blade-B2, and Blade-B3 was observed with R^2^ values of 0.90, 0.82, and 0.88, respectively. Thus, the developed model for predicting cutting power was adequate for all three blades^26^.

### Effect of blade types

The effect of all three blades on cutting force and power requirement was studied with a paired ‘t’ test. It was applied for the comparison of three blades in terms of maximum cutting force and power requirement. Table 6 revealed that the cutting force and the maximum power required for all three blades significantly differed at a 1% significance level.

**Table 6:** Selection of cutter bar blade.

Cutter bar blade	Pitch (mm)	Bevel angle (°)	Minimum cutting force (N)	Maximum cutting force (N)	Maximum power requirement (W)
Blade-B1	22	22.5	15.96^a^	58.97^a^	105.25^a^
Blade-B2	25	25.0	21.08^b^	76.63^b^	124.60^b^
Blade-B3	28	28.0	30.21^c^	85.31^c^	136.84^c^

Numerical in subheads having different alphabetic superscripts are statistically different.

Table 7 depicted that the cutting force and cutting power for all the pairs of cutter bar blades (i.e., Blade-B1 & Blade-B2, Blade-B2 & Blade-B3 and Blade-B1 & Blade-B3) are significantly different from each other. Figure 12 shows that initially, the power requirements for all three blades were nearly equal, but at higher cutter bar speeds, the power requirements had significant differences for each blade. Blade-B1 required 18 and 30% less power than Blade-B2 and Blade-B3, respectively. Therefore, the cutter bar Blade-B1 can be appropriate for the development of the harvester as it requires minimum cutting force and power to cut the cumin plants.

**Table 7:** Statistical comparison of the three cutter bar blades.

Experiments		Cutting force			Cutter bar power		
Pair	Blade	t	df	Sig. (2-tailed)	t	df	Sig. (2-tailed)
Pair 1	Blade-B1 & Blade-B2	− 24.87	2	0.002	− 118.02	2	0.0001
Pair 2	Blade-B2 & Blade-B3	− 33.01	2	0.001	− 96.30	2	0.0001
Pair 3	Blade-B1 & Blade-B3	− 189.12	2	0.001	− 119.23	2	0.0001

## Discussion

Results revealed that the cutting force followed a decreasing trend with the increase in cutter bar speed for all three blades. It might be because, at low cutting speed, the stalks tend to get flattened and crushed, resulting in a significant resistive force during the cutting process^24,26,47^. A similar finding was observed by Sushilendra et al.^53^ for chickpea stem and Kumawat and Rehman^25^ for onion leaves. Sarkar and Rehman^7^ also concluded that the less resistance offered by the stem at higher cutter bar speeds for cabbage stem. However, Song et al.^43^ suggested that crop materials are viscoelastic composite materials that undergo two stages i.e., compression deformation and fracture during the cutting process. In compression deformation, the fibers are extruded and deformed; this process is a function of time. Thus, the compression deformation time is reduced at higher cutter bar speeds, leading to less cutting force. This was in agreement with the findings of Wang et al.^54^. It was also observed that the cutting force followed a linearly increasing trend with the increase in forward speed. It might be due to the increase in forward speed^51^. A greater number of plants get cut per unit of time, resulting in an increase in cutting force. Similar findings were reported by Sahoo and Raheman^26^ and Modak and Rehman^27^. For paddy stem. Kumawat and Rehman^25^ also concluded that with the increase in forward speed, the cutting material handled per unit of time increased cutting resistance, resulting in higher cutting force. The cutting force was highest for the cutter bar Blade-B3 compared to the other two blades, Blade-B1 and Blade-B2. It might be due to the larger pitch and bevel angle of the Blade-B3 cutter bar^55–59^. Wang et al.^54^ also reported that with an increase in the pitch of the blade, the cutting force increased^37,38,60–71^. The power requirement was increased with the increase in cutter bar speed. It might be due to the increased idle cutting power with cutter bar speed. The idle cutting power played a major role in total power consumption for cutting. Vu et al.^44^ and Sessiz et al.^72^ also reported similar results, as high cutting velocity increases power consumption for cutting stems^73,74^.

## Conclusion

Based on this study, the following conclusions were drawn:

- Quadratic and linear models effectively described cutting force across different blades. The lack of fit tests and a high coefficient of determination values confirmed model adequacy. Cutter bar speed had the most significant impact on cutting force, followed by forward speed. Quadratic terms of these variables also contributed, albeit to a lesser extent.
- The cutting force decreased with an increase in the cutter bar speed for all three blades. Similarly, it was observed that the cutting force followed a linearly increasing trend with an increase in forward speed. Blade-B3 exhibited the highest cutting force due to its larger pitch and bevel angle. Power requirement increased with cutter bar speed primarily due to higher idle cutting power.
- The maximum cutting force for Blade-B1, Blade-B2 and Blade-B3 were 58.97, 76.63 and 85.31 N respectively. The total power requirement for cutting cumin crops by Blade-B1, Blade-B2, and Blade-B3 were 105.25, 124.60, and 136.84 W, respectively.
- Mathematical models accurately depicted the relationship between cutter bar speed, forward speed, and cutting force for each blade, with high coefficient of determination values (0.95 to 0.97) confirming their validity.
- The cutter bar Blade-B1 can be appropriate for the cumin harvester. It exhibited lower cutting force (15.96 to 58.97 N) and power requirement (105.25 W) than Blade-B2 and Blade-B3.

## Data Availability

The datasets used and/or analysed during the current study available from the corresponding author on reasonable request.
